# A Novel Brominated Alkaloid Securidine A, Isolated from the Marine Bryozoan *Securiflustra securifrons*

**DOI:** 10.3390/molecules22071236

**Published:** 2017-07-23

**Authors:** Priyanka Michael, Kine Ø. Hansen, Johan Isaksson, Jeanette H. Andersen, Espen Hansen

**Affiliations:** 1 MARBIO, UiT–The Arctic University of Norway, Breivika, Tromsø N-9037, Norway; jeanette.h.andersen@uit.no (J.H.A.); espen.hansen@uit.no (E.H.); 2 Department of Chemistry, UiT–The Arctic University of Norway, Breivika, Tromsø N-9037, Norway; johan.isaksson@uit.no

**Keywords:** secondary metabolites, marine invertebrates, marine bryozoans, *Securiflustra securifrons*, Securidine A, biological activity

## Abstract

A novel brominated alkaloid, Securidine A, was isolated from the cold water marine bryozoan *Securiflustra securifrons*. Securidine A was isolated using semi-preparative HPLC, and the structure was elucidated by spectroscopic methods. The isolated Securidine A was tested for cytotoxic, antibacterial, and anti-diabetic activities as well as for its potential for inhibition of biofilm formation. No significant biological activity was observed in the applied bioassays, thus expanded bioactivity profiling is required, in order to reveal any potential applications for Securidine A.

## 1. Introduction

Marine organisms are considered to be a rich source for natural products, and currently almost 50,000 marine natural products are reported in the Dictionary of Marine Natural Products [1]. A statistical analysis of marine natural products discovered between 1985 and 2008 estimates that approximately 75% of the compounds are isolated from sessile and soft-bodied invertebrates including sponges, ascidians, cnidarians, mollusks and bryozoans [2,3]. In order to protect themselves from predators and to compete for space and food, many invertebrates have evolved the ability to produce secondary metabolites as a chemical defense, which increase their ability to survive, grow, and reproduce [4,5]. Bryozoans, or moss animals, are aquatic invertebrates that belong to the phylum Bryozoa (Polyzoa). They are sedentary and filter feeders. These colonial organisms consist of tiny intercommunicating individuals called zooids [6]. Bryozoans are widely distributed in tropical and temperate marine waters [7], and are common fouling organisms on marine substrates [6]. The phylum consists of 3 classes, 4 orders, 187 families, and 808 genera, and approximately 8000 species are described, most of them are observed in marine habitats [8,9]. Marine bryozoans are known to produce a significant number of biologically active secondary metabolites, including macrolides, alkaloids, sterols, and heteroatom-containing compounds [3,10] that possess antitumor [11], antibacterial [12,13], and muscle relaxant activities [14]. The well-known bioactive compound bryostatin 1 is a macrolide isolated from the marine bryozoan *Bugula neritina*, and it is proven to be an antineoplastic agent [15]. Secondary metabolites isolated from marine bryozoans have been extensively reviewed [6,8,16,17].

**Figure 1:**
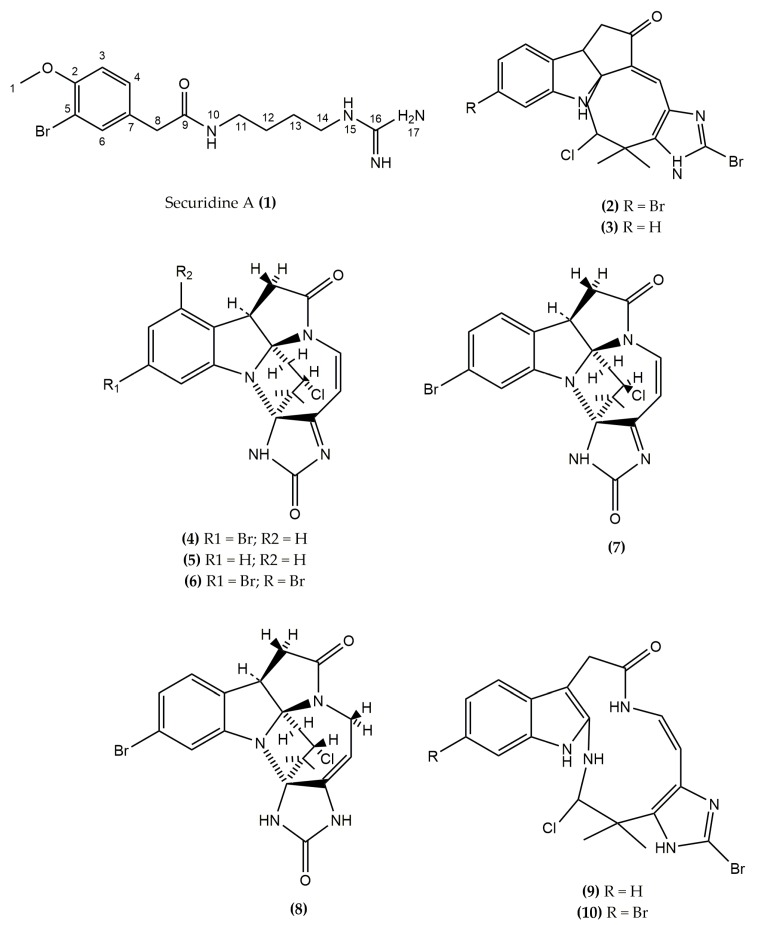
Structure of novel compound Securidine A (**1**), Securamine A–G (**2**–**8**), and Securines A and B (**9**–**10**).

In the present study, we isolated the novel mono brominated tyrosine derivative Securidine A (**1**) from the marine bryozoan S*ecuriflustra securifrons. S. securifrons* (Pallas, 1766) is a temperate and subtidal species abundant in the North Sea [18,19], and the encrusting basal portion of the colonies are attached to hard substrates such as stones and shells. In previous reports, seven halogenated indole-imidazole alkaloids, Securamines A–G and Securines A and B (obtained by dissolving Securamines A and B in DMSO-*d*_6_), have been isolated from *S. securifrons*, but no biological activities were recorded for any of these compounds. The halogenated Securamines A–G (Figure 1) differ from each other by the presence and absence of a bromine substitution or a number of bromine substitutions in the benzene ring. [20,21]. These compounds are structurally similar to chartellines A–C and chartellamides A and B, isolated from another marine bryozoan *Chartella papyracea*. Both species belong to the Flustridae family (Order: Cheilostomata). Many of the Cheilostome bryozoans including *S. securifrons*, *C. papyracea*, and *Flustra foliacea* are producing indole alkaloids and brominated alkaloids [12,20,21,22,23,24,25,26,27,28,29,30,31,32]. However, the isolated new brominated alkaloid, Securidine A (**1**) is a novel structure (Figure 1), which is not structurally similar to any other listed compounds of *S. securifrons.*

The current paper describes the isolation of new compound Securidine A (**1**), its structural characterization and potential biological activities were also investigated.

## 2. Results and Discussion

The marine bryozoan *S. securifrons* was collected in April 2014 in the North Sea. Securidine A (**1**) was isolated from the aqueous extract of *S. securifrons* through stepwise fractionation. The lyophilized animal material was ground and extracted with ultrapure water. The freeze-dried aqueous extract was pre-fractionated using column chromatography with a Biotage SP24 HPFC flash purification system. The extract was eluted with a gradient of H_2_O/CH_3_OH/CH_3_-CO-CH_3_ and the fractions were assayed for cytotoxicity against melanoma (A2058) and colon adenocarcinoma (HT29) cell lines using an MTS based anticancer assay. Flash fractions four and five showed cytotoxic activity. UPLC-HR-MS analysis of these fractions, followed by isotopic pattern examination of the eluting compounds, revealed the presence of a mono brominated compound. For the isolation, the aqueous extract was injected onto semi-preparative RP-HPLC C_18_ column and Securidine A (**1**) was eluted with a 10 minutes gradient, changing from 10% to 40% CH_3_CN over 10 minutes. For the second purification step, a Phenyl-Hexyl prep HPLC column was used with a 10 min gradient, changing from 10% to 34% CH_3_CN.

The HR-MS analysis suggested that Securidine A (**1**) contained a bromine. The molecular formula was determined to be C_14_H_21_BrN_4_O_2_, corresponding to the protonated molecular peak at *m*/*z* 357.0858 ([M + H]^+^, calculated *m*/*z* 357.0926), suggesting six degrees of unsaturation. A database search indicated that the isolated compound was novel, and its structure was solved based on a number of 1D and 2D NMR experiments.

**Figure 2:**
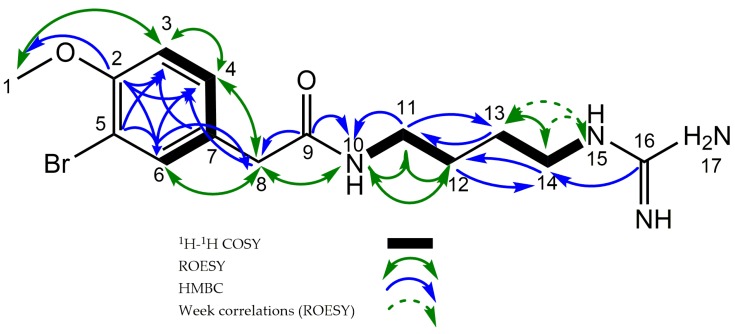
Key HMBC, ME-HSQC, H2BC, COSY, and ROESY correlations of Securidine A (**1**).

Interpretation of the available 1D ^1^H (presat, 1DNOE and excitation sculpting), 1D ^13^C, HMBC, ME-HSQC, H2BC, COSY, and ROESY (Supplementary materials Figures S1 to S6) led to an unambiguous structural elucidation of **1**. Examination and comparison of the 1D ^13^C, HSQC, and HMBC showed that **1** had 14 carbons consisting of one methyl group, five methylenes, three methines, and five quaternary carbon atoms. Two ^1^H resonances, H-10 and H-15, were identified as NH groups based on the absence of any ^1^*J*_CH_, broadened line widths and deshielded chemical shifts. Three methines indicated the presence of a disubstituted phenyl ring attached to the main chain. The methyl group protons (δ_H_ 3.84) showed HMBC correlation to the quaternary C-2 (δ_C_ 156.2) as well as ROESY correlation to the H-3 (δ_H_ 6.96) methine proton. The relatively deshielded chemical shift value of the methyl protons (δ_H_ 3.84) was assigned as the protons of a methoxy group. The high chemical shift value of C-2 (δ_C_ 156.2) further confirmed this. COSY and ROESY correlations between H-3 (δ_H_ 6.96) and H-4 (δ_H_ 7.22) as well as a shared coupling constant of 8.6 between the two, places C-3 and C-4 next to each other. Additionally, the H-3 methine showed HMBC correlation to a halogenated quaternary carbon (C-5, δ_C_ 112.2), the quaternary C-7, (δ_C_ 130.5) as well as to the final methine carbon (C-6, δ_C_ 134.5). The distinction between C-7 and C-5 was made based on their chemical shift values and a ^2^*J_CH_* from C-5 to H-6, which is commonly observed from halogenated carbons but not from carbons in phenyl rings further away from heteroatoms. The H-8 (δ_H_ 3.41) methylene showed ROESY and HMBC correlations to the protons and carbons in position 4 (δ_H_ 7.22, δ_C_ 130.2) and 6 (δ_H_ 7.47, δ_C_ 134.5), placing it in ortho position to C-4 and C-6 in the phenyl ring. The phenyl group was thus methoxy-substituted in the para position and brominated in the meta position, while and the main chain of the molecule was attached to C-7. The H-8 (δ_H_ 3.41) protons further showed a HMBC correlation to a deshielded quaternary carbon (C-9, δ_C_ 173.7), characteristic for a carbonyl carbon. HMBC correlations were observed to H-10 (δ_H_ 8.20) from C-9 and to H-10 from C-11 (δ_C_ 39.8). COSY and ROESY correlations were observed between the methylene groups in the 11 (δ_H_ 3.20, δ_C_ 39.8) and 12 positions (δ_H_ 1.56, δ_C_ 27.5). The same correlations were observed between the methylene groups in position 13 (δ_H_ 1.55, δ_C_ 27.0) and 14 (δ_H_ 3.16, δ_C_ 42.0). The methylene groups in the 12 and 13 position were near isochronous, but could be distinguished by their respective couplings to H-11 and H-14 in H2BC and COSY. H-14 further correlated with the quaternary C-16 (δ_C_ 158.8) in HMBC spectra. H-13 (δ_H_ 1.55) and H-14 (δ_H_ 3.16) both showed ROESY correlations to H-15 (δ_H_ 7.57). This, in addition to a COSY correlation between H-14 and H-15, places a NH-group in the 15 position. Thus, the remaining part of the chemical formula was N_2_H_3_, leaving the only possible structural solution to be the attachment of an NH- and an NH_2_- group to C-16. The deshielded shift value of C-16 (δ_C_ 158.8) was in good agreement with this conclusion (Table 1). Selected 2D correlations utilized in determining the structure of **1** are shown in Figure 2. The compound was named Securidine A.

### Bioactivity

The pure Securidine A (**1**) was evaluated for its biological activity in a selection of bioassays including anti-cancer and anti-bacterial activity, inhibition of protein tyrosine phosphatase 1B (PTP-1B) for anti-diabetic activity, as well as for inhibition of biofilm formation.

Securidine A (**1**) was assessed for its cytotoxicity against three cancer cell lines (A2058 melanoma, HT29 colon adenocarcinoma and MCF7 breast carcinoma) by the MTS method, since flash fractions of the extract from *S. securifrons* showed anticancer activity against A2058 melanoma and HT29 colon adenocarcinoma cancer cell lines in the MabCent screening program [33]. However, the isolated compound, Securidine A (**1**), did not show any significant activity against the cancer cell lines at 50 µM (17.87 µg/mL) or 100 µM (37.73 µg/mL). Securidine A (**1**) was also tested for its antibacterial activity, and no activity was seen against *Staphyloccoccus aureus*, *Escherichia coli*, *Enterococcus faecalis*, *Pseudomonas aeruginosa*, or *Streptococcus agalactiae*.

**Figure 3:**
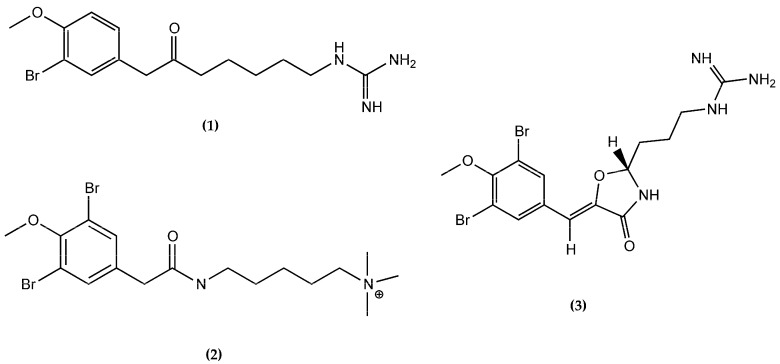
The structurally related compounds Securidine A (**1**), Pulmonarin (**2**), and Synoxazolidinone B (**3**).

Securidine A (**1**) is a brominated tyrosine derivative. It is structurally related to two other brominated tyrosine derivatives, Pulmonarin B (**2**) and Synoxazolidinone B (**3**) (Figure 3), both isolated from the sub-Arctic ascidian *Synoicum pulmonaria* [34,35]. Pulmonarin B (**2**) contains a quaternary ammonium group in the side-chain instead of the guanidine group of Securidine A (**1**) and Pulmonarin B (**2**) did not have any antibiotic or cytotoxic activity. Both Securidine A (**1**) and Synoxazolidinone B (**3**) have a guanidine group in the side chain, but Synoxazolidinone B (**3**) has a central 4-oxazolidinone core, linking a di-brominated tyrosine derivative to an arginine [36]. Synoxazolidinone B (**3**) is active against the Gram-positive bacteria, methicillin resistant *S. aureus*. Macherla et al. suggested that a 4-oxazolidinone core is a good pharmacophore for antibacterial activity [36]. The difference between Securidine A (**1**) and Synoxazolidinone B (**3**) in activity against bacteria might thus be explained by the lack of a central 4-oxazolidinone core in Securidine A (**1**). Moreover, most of the antimicrobial peptides or alkaloids are amphipathic molecules, including Synoxazolidinone B, which displays two cationic groups and two bulky lipophilic groups in order to fulfill the structural requirements of antibacterial activity [37,38,39]. A study of the pharmacophore of small antibacterial peptides demonstrated that one cationic group as well as a minimum of two bulky groups were required for activity against Gram-positive staphylococci [40].

The effect of Securidine A (**1**) against PTP-1B was also examined, since PTP-1B is regarded as a promising target for the treatment of diabetes [41]. However, no inhibition of PTP-1B was observed at 50 µM or 100 µM. Many potent antifouling compounds are brominated tyrosine derivatives that have cationic guanidine or guanidine-like groups [42]. Nevertheless, no inhibition of biofilm formation by *Staphylococcus epidermidis* was found at 100 µM.

## 3. Materials and Methods 

### 3.1. Animal Material

The marine bryozoan *Securiflustra securifrons* (Palllas, 1766) was collected by an Agassiz trawl at 73 m depth from west Spitzbergen (71.0759° N, 24.4355° E) in April 2014. The fresh sample weighed 336.01 g and was frozen immediately at −23 °C. The specimen was identified by Norwegian marine biobank Marbank (Institute of Marine Research, Tromsø, Norway) and they have a taxonomical reference sample in their collection. The sample was stored at −23 °C until further processing.

### 3.2. Extraction

The lyophilized animal material was ground and extracted twice with ultrapure water (Milli-Q; Millipore, Billerica, MA, USA) at 4 °C for 24 h in darkness. After centrifugation (4000 rpm at 5 °C for 30 min), the supernatants were pooled and reduced under reduced pressure at 40 °C, yielding 24.78 g of aqueous extract. The remaining pellet was freeze-dried and extracted twice with 1:1 *v*/*v* of dichloromethane (Merck, Darmstadt, Germany) and methanol (MeOH; Sigma-Aldrich, Steinheim, Germany) at 4 °C. The combined organic extract was subsequently filtered through a Whatman No. 3 filter paper (Little Chalfont, UK) and concentrated under reduced pressure, affording 5.13 g of organic extract. Both extracts were stored at −23 °C until further use. Aqueous extract was used for isolation of Securidine A (**1**), in order to avoid including lower polarity molecules in the purification process.

### 3.3. Isolation and Purification of Compound **1**

An aliquot of the lyophilized aqueous extract (1.5 g) was mixed with 90% MeOH (6–8 mL), cooled to 4 °C, and centrifuged at 4000 rpm and 4 °C, in order to precipitate salt. The supernatant was mixed with 3.0 g resin (Dianon ® HP-20, Supelco Analytical, Bellefonte, PA, USA) and the mixture was dried by SpeedVac. The dried resin loaded with the aqueous extract was deposited on top of a column pre-packed with 6.5 g HP20SS, and the extract eluted from the column using a gradient of H_2_O/CH_3_OH/CH_3_COCH_3_. The pre-fractionation was performed using a Biotage SP4 chromatography HPFC flash purification system (Biotage, Charlottesville, NA, USA), resulting in eight fractions. These eight fractions were assayed for cytotoxicity against A2058 melanoma and HT29 colon adenocarcinoma cell lines using the MTS based anticancer assay. Fractions four and five showed cytotoxic activity and these active fractions were de-replicated using HR-MS. Securidine A (**1**) was isolated directly from the aqueous extract by mass guided prep-HPLC.

Isolation of Securidine A (**1**) was performed by a Waters HPLC purification system. The system was composed of a 600 pump, a 2996 photodiode array UV detector, a 3100 mass detector and a 2767 sample manager (Milford, MA, USA). The system was controlled with Waters MassLynx version 4.1 and the FractionLynx application manager. The mass of Securidine A (**1**) (*m*/*z* 356.2520) was used as the collection trigger. The aqueous extract was injected onto a Waters Xterra RP18 (10 mm × 300 mm, 10 µm) HPLC column. Securidine A (**1**) was eluted with a 10 min with a gradient from 10% to 40% CH_3_CN (Chromasolv; Sigma-Aldrich) in ultrapure water, both containing 0.1% formic acid (Pro-analysis, Merck), and the flow rate was 6 mL/min. Further purification was done by Waters Phenyl-Hexyl Prep (10 mm × 250 mm, 5 µm) HPLC column with a gradient from 10% to 34% of CH_3_CN (Chromasolv; Sigma-Aldrich) in ultrapure water with a 10 min and the flow rate of 6 mL/min.

### 3.4. Structure Determination

UPLC-HR-MS analysis and NMR were used to confirm the structure of Securidine A (**1**). UPLC-HR-MS analysis was performed on an Acquity UPLC and a LCT premier Time-of-Fight MS using ESI^+^ ionization (Waters). MassLynx version 4.1 was used to process the MS data. The NMR experiments were acquired on a Bruker Avance III HD spectrometer operating at 599.90 MHz for protons, equipped with an inverse detected cryo-probe enhanced for ^1^H, ^13^C, and ^2^H. The NMR samples were prepared by dissolving Securidine A (**1**) in 500 µL methanol-*d*_3_ and transferred into a 5 mm disposable tube. Experiments were typically acquired using gradient selected adiabatic versions where applicable. All experiments were acquired using Top Spin 3.5 pl2 at 298 K.

### 3.5. Bioassays

#### 3.5.1. Cytotoxicity

*Cell culturing*—Human melanoma A2058 (ATCC-CRL-1114 TM, Manassas, VA, USA) and human colon adenocarcinoma HT29 (ATCC HTB-38 ^TM^) were grown in Dulbecco’s Modified Eagle Medium (DMEM) (Life technologies ^TM^, city, UK) and Roswell Park Memorial Institute (RPMI)–1640 medium (Biochrome GmbH, Germany) respectively. Both media were supplemented with fetal bovine serum (10%), gentamycin (10 mg/ml), and l-alanyl-l-glutamine (200 mM). Human breast cancer MCF7 (ATCC HTB-22 ^TM^) cells were grown in Eagle’s Minimum Essential Medium (E-MEM) (Biochrom, GmbH, Berlin, Germany) supplemented with fetal bovine serum (10%), gentamycin (10 mg/mL), non-essential amino acid without l-alanyl-l-glutamine, sodium pyruvate (100 mM), and sodium bicarbonate (7.5% *w*/*v*). The supplements were purchased from Biochem, GmbH, Berlin, Germany. All cells were incubated at 37 °C with 5% CO_2_ and 95% air. Cells were sub-cultured with fresh medium every three to four days. The test concentrations (50 µM and 100 µM) were prepared from the stock solution of pure compound (10 mg/mL in 100% DMSO) which is dissolved in supplemented RPMI-1640 medium.

Cell viability was tested against three adherent cancer cell lines, human melanoma A2058, human colon adenocarcinoma HT29, and human breast cancer MCF7 cell lines in vitro, using an MTS [3-(4,5-dimethylthiazol-2-yl)-5-(3-carboxymethoxyphenyl)-2-(4-sulfophenyl)-2*H*-tetrazolium inner salt] colorimetric method. The growing cells were suspended in 100 µL of supplemented medium seeded at a density of 2000 cells/well in a 96-well microtiter plates (Nunclon ^TM^ Delta Surface, Thermo Fisher Scientific, Denmark). The cells were incubated at 37 °C for 24 h. After removal of old medium, the cells were treated with 50 µM and 100 µM of Securidine A (**1**). After 72 h incubation, 10 µL of Cell titer 96 ^®^ Aqueous One Solution Reagent (Promega, Madison, WI, USA) was added to each well and the plates were incubated for 1 h. The absorbance readings at 485 nm was measured using DTX 880 multimode detector (Beckman coulter, CA, USA). Cells with RPMI-1640 medium were used as negative control and cells treated with Triton^®^ X-100 (Sigma-Aldrich, Steinheim, Germany) as positive control. Growth inhibition was measured by optical density (OD) and was calculated as: Cell survival (%) = (Average of parallels – positive control)/(negative control – positive control) × 100

#### 3.5.2 Antibacterial Assay

*Test strains and culture media*—The antibacterial activity of Securidine A (**1**) was determined by using a minimum inhibitory concentration (MIC) assay against *Staphylococcus aureus* (ATCC 25923), *Escherichia coli* (ATCC 25922), *Enterococcus faecalis* (ATCC 29212), *Pseudomonas aeruginosa* (ATCC 27853), and *Streptococcus agalactiae* (ATCC 12386). Antibacterial test was performed in sterile Muller Hinton broth (Becton Dickinson, Sparks, MD, USA) and brain heart infusion broth (Becton Dickinson). The bacterial cultures were obtained from the stock (−80 °C) and were grown on blood agar (acquired from the University hospital, UiT, Tromsø, Norway) at 37 °C for 24 h and working bacterial stock culture were maintained at 4 °C. An overnight culture of each strain was prepared and 2 mL of overnight culture was inoculated in 25 mL of growth media and incubated at 37 °C on the shaker at 180 rpm, until the culture reaches the turbidity according to 0.5 McFarland standard (1.0 × 10^8^ colony forming unit (CFU)/mL). In this study, bacterial cultures were diluted in 1:100 and then 1:10 in growth media and the final concentration of bacterial cells in the wells were adjusted to 0.5–3.0 × 10^5^ (CFU)/mL of *S. aureus*, *E. coli*, *E. faecalis*, and *Streptococcus agalactiae* and 3.0–7.0 × 10^4^ CFU/mL of *P. aeruginosa*. Test concentrations (50 µM and 100 µM) were prepared from the stock solution of Securidine A (**1**) (10 mg/mL in 100% DMSO), and was dissolved in MilliQ water.

From the test concentrations, 50 µL was transferred into a 96-well microtiter plate (Nunclon^TM^ Delta Surface, Thermo Fisher Scientific, Denmark). Subsequently, 50 µL of final concentration of bacterial cells were added and incubated at 37 °C for 24 h. After incubation, the activity was measured as absorbance at 600 nm in a plate reader (1420 Victor^3 TM^ multilabel counter, PerkinElmer^®^, Singapore) and WorkOut 2.0 software (Dazdaq Ltd, Brighton, UK) was used for plate reading. Bacterial suspension with MilliQ water was used as positive control and growth medium with MilliQ water as negative control. Gentamycin (Amresco, OH, USA) was used as the reference control of this assay. The inhibition was evaluated by the average of parallel OD value. The OD value ≤0.05 considered as active and ≥0.09 was considered inactive.

#### 3.5.3 Protein Tyrosine Phosphatase 1B Assay

*Reagents*—(4-(2-hydroxyethyl)-1-piperazineethanesulfonic acid (HEPES; Sigma-Aldrich, Steinheim, Germany), sodium chloride (NaCl; Sigma-Aldrich), dithiothreitol (Sigma-Aldrich), ethylene diamine tetra-acetic acid disodium salt dehydrate (EDTA; Sigma-Aldrich), bovine serum albumin (Sigma Aldrich), human recombinant protein tyrosine phosphatase 1B enzyme (PTP1B; Merck-Calbiochem, Darmstadt, Germany), protein tyrosine phosphatase inhibitor IV (Merck-Calbiochem), 6,8-difluoro-4-methylumbelliferylphosphate (DiFMUP; VWR, Leuven, Belgium).

The test concentration (10 µM and 50 µM) was obtained from stock solution (10 mg/mL in 100% DMSO) of Securidine A (**1**) and was dissolved in the assay buffer.

PTP1B activity of Securidine A (**1**) was measured in the presence of human recombinant PTP1B enzyme, flurogenic substrate DiFMUP, and the buffer solution in black polystyrene 96-well microtiter plate (FluoroNunc^TM^ MaxiSorp Surface, Capital Scientific, TX, USA). The final volume of the mixture was 100 µL in each well. Each well contained 25 µL of buffer solution (pH 7.2) (25 mM HEPES, 50 mM NaCl, 2 mM dithiothreitol, 2.5 mM EDTA and 0.01 mg/mL bovine serum albumin), 25 µL of Securidine A (**1**), and 50 µL of PTP1B enzyme (31.2 ng/mL). Then, the plate was incubated at 37 °C. After 30 min incubation, 25 µL of 10 µM fluorogenic substrate DiFMUP was added into wells and incubated again at 37 °C for 10 min. The inhibition of enzyme activity was measured as the intensity of fluorescence at 360 nm excitation and 465 nm emission wavelengths by using DTX 880 Multimode detector (Beckman coulter, CA, USA). The assay buffer was used as the positive control and protein tyrosine inhibitor IV (160 µM) was used as the negative control. The inhibition of enzyme activity was measured from the fluorescence and calculated with positive and negative control. The inhibition of enzyme activity was expressed in percentage and the activity lower than 30% was considered active. The inhibition of enzyme activity was calculated by (Average of triplicates – negative control)/(positive control – negative control) × 100.

#### 3.5.4. Biofilm Assay

*Test strains*—The test strains *Staphylococcus epidermidis* (RP62A-42-77 ATCC-35984) and *Staphylococcus haemolyticus* (Clinical isolate 8-7A) from the stock were grown on blood agar (acquired from University hospital, UiT, Tromsø) at 37 °C for 24 h and these working bacterial stock were maintained at 4 °C.

The bacterial strain *S. epidermidis* was used to test the biofilm formation on the polystyrene 96-well microtiter plate (Nunclon ^TM^ Delta Surface, 734-2097, Thermo Fisher Scientific, Denmark). The overnight culture was grown in 5 mL of sterile tryptic soy broth (TSB; Merck, Darmstadt, Germany) in a shaking incubator at 37 °C and diluted in 1:100 in TSB medium with 1% of glucose (Sigma-Aldrich). Securidine A (**1**) from the stock (10 mg/mL) was dissolved in the MilliQ water, to prepare 100 µM test concentration. From the test concentration, 50 µL was transferred into the wells, subsequently the diluted test strain 50 µL was added and incubated at 37 °C for overnight. *S. epidermidis* with sterile MilliQ water was used as positive, *S*. *haemolyticus* with sterile MilliQ water was used as negative control and medium, with sterile MilliQ water used as blank.

The biofilm formation in the well was determined by using crystal violet (Merck) staining. The bacterial suspension was removed carefully on the cell paper and the plate was gently washed with tap water two or three times. After the biofilm fixation was done at 65 °C for 1 h, the cells were stained with 70 µL 0.1% of crystal violet. After 10 min incubation, the crystal violet was removed carefully on the cell paper and the plate was washed with tap water two or three times. The plate was dried at 65 °C for 1 h and 70 µL 70% ethanol (VWR) was added into the wells. Thereafter, the microtiter plate was placed on a shaker for 5–10 min. The inhibition of biofilm was assessed by using in the Victor^3 TM^ multilabel counter at 600 nm and measuring the optical density (OD). If the inhibition of biofilm rate was 0.25 or lower the OD value was considered active.

## 4. Conclusions

The new brominated tyrosine derivative Securidine A (**1**) was isolated from the aqueous extract of the marine bryozoan *Securiflustra securifrons*. The structure was determined by interpretation of data from 1D and 2D NMR experiments and mass spectrometry analysis. In this study, Securidine A (**1**) did not show any significant biological activities in the applied bioassays. Further bioactivity profiling is required in order to identify any potential biological activities of the molecule.

## Figures and Tables

**Table 1:** NMR spectroscopic data ^a^ (600 MHz, methanol-*d*_3_).

Position	δ_C_, Type	δ_H_ (*J* in Hz)	COSY	^1^H, ^13^C-HMBC ^b^	^1^H, ^13^C H2BC	ROESY
1	56.6, CH_3_	3.84, s		2		3
2	156.2, C					
3	113.1, CH	6.96, d (8.6)	4	2,5,7	4	1,4
4	130.2, CH	7.22, dd (8.6, 2.3)	3,6	2,3,6,8	3	3,8
5	112.2, C					
6	134.5, CH	7.47, d (2.3)	4	2,4,5,8		8
7	130.5, C					
8	42.4, CH_2_	3.41, s		4,6,9		4,6,10
9	173.7, C					
10		8.20, s	11	9,11	11	8,11,12 ^w^
11	39.8, CH_2_	3.20, q (6.4)	10,12	9,(12/13) ^d^	12	10,12
12	27.5, CH_2_	1.56, m ^c^	11	(11/14) ^d^	11	10 ^w^,11
13	27.0, CH_2_	1.55, m ^c^	14	(11/14) ^d^	14	14,15 ^w^
14	42.0, CH_2_	3.16, m ^c^	13,15	16,(12/13) ^d^	13	13,15 ^w^
15		7.57, s	14		14	13 ^w^,14 ^w^
16	158.8, C					

^a 1^H 1D, ^13^C, ^1^H, ^1^H-COSY, ^1^H,^13^C-HMBC (full and band selective) spectra are included in the supplementary data, Figures S1–S6 (Supplementary materials). ^b 1^H 1D, ^13^C-HMBC correlations are from the proton(s) stated to the indicated carbon; ^c^ Overlapping and/or broadened peaks impeding complete multiplet analysis. Chemical shift extracted from ^1^H, ^13^C-HSQC, ^1^H, ^13^C-HMBC and/or ^1^H, ^13^C-H2BC; ^d^ ambiguous correlations in HMBC, C12 and C13 assignment based on H2BC correlations (Figure S4); ^w^ Weak correlations.

## Supplementary Materials

Supplementary data S1–S6 (^1^H, ^13^C, HSQC, HMBC, H2BC, COSY and ROESY NMR spectral data of isolated compound) associated with this article can be found online.
