# Supplementary material for: A Novel Brominated Alkaloid Securidine A, Isolated from the Marine Bryozoan Securiflustra securifrons
Source: Molecules. 2017 Jul 23;22(7):1236. doi: 10.3390/molecules22071236 (PMC6152195; doi:10.3390/molecules22071236)
Supplement: Supplementary file 1 [file molecules-22-01236-s001.pdf]

Title: A Novel Brominated Alkaloid Securidine A, Isolated from the Marine Bryozoan *Securiflustra securifrons*

Supplementary materials: 1D NOE, 13C-NMR, HMBC, ME-HSQC, H2BC, COSY and ROESY correlations of compound Securidine A **(1)**

Authors: Priyanka Siranjeevi [1*a], Kine Ø. Hansen [1*a], Johan Isaksson [2], Jeanette H. Andersen [1] and Espen Hansen [1,]

[1] MARBIO, UiT – The Arctic University of Norway, Brevika, N-9037, Tromsø, Norway; raja.p.siranjeevi@uit.no (P.S); kine.o.hanssen@uit.no (K.Ø.H); jeanette.h.andersen@uit.no (J.H.A); espen.hansen@uit.no (E.H)

[2] Department of Chemistry, UiT – The Arctic University of Norway, Brevika, N-9037, Tromsø, Norway; johan.isaksson@uit.no (J.I)

***** Correspondence: raja.p.siranjeevi@uit.no; Tel.: +47-77649268; kine.o.hanssen@uit.no; Tel.: +47-77649272

[a] These authors contributed equally to this work.

Figure S1. 1D NOE spectrum of securidine A (**1**)

Figure S2. 13C-NMR spectrum of **1**.

Figure S3. Combined HSQC and HMBC spectra of **1**.

Figure S4. H2BC spectra of **1**

Figure S5. COSY Spectrum of **1**.

Figure S6. ROESY spectrum of **1**.

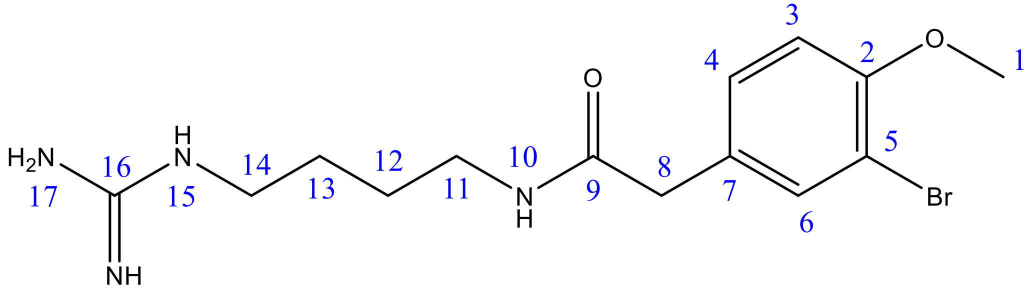

Chemical Formula: $C_{14}H_{21}BrN_4O_2$
Exact Mass: 356,08

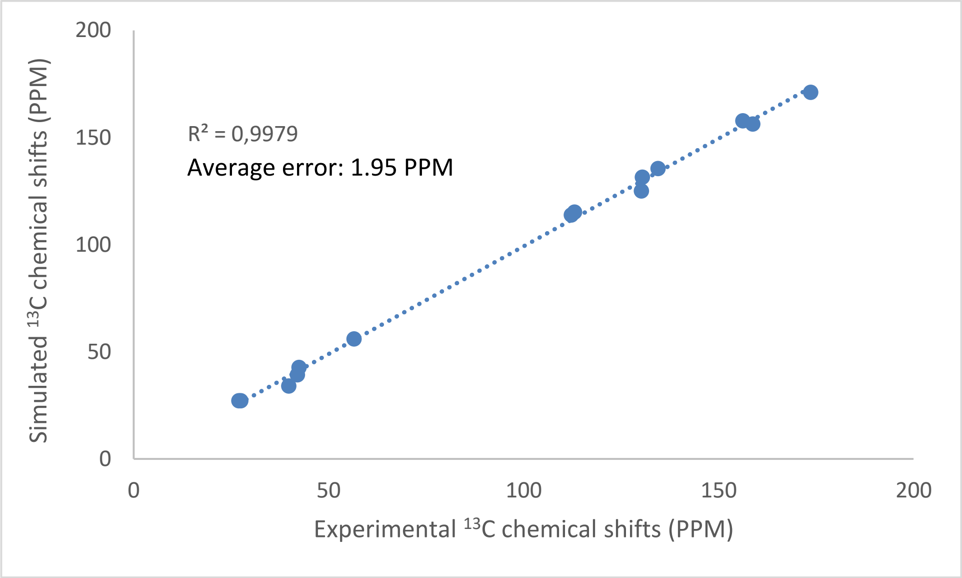

^{1}H NMR (600 MHz, Methanol-d_4) δ 8.20 (s, 1H), 7.57 (s, 1H), 7.47 (d, J = 2.3 Hz, 1H), 7.22 (dd, J = 8.6, 2.3 Hz, 1H), 6.96 (d, J = 8.8 Hz, 1H), 3.84 (s, 3H), 3.41 (s, 4H), 3.20 (q, J = 6.4 Hz, 2H), 3.18 – 3.10 (m, 2H), 1.60 – 1.49 (m, 4H).

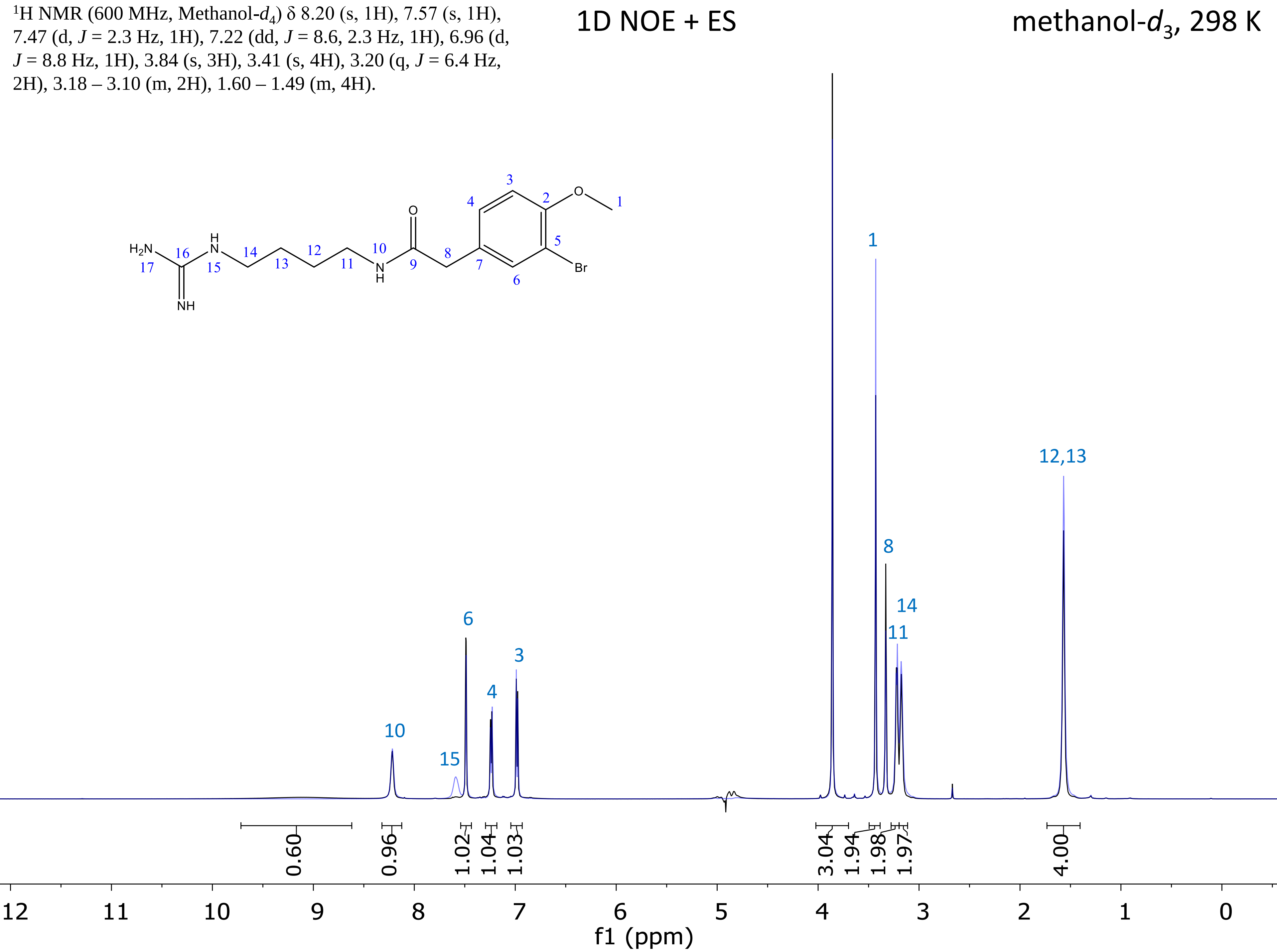

^{13}C NMR (151 MHz, MeOD) δ 173.96, 158.77, 156.25, 134.52, 130.48, 130.25, 113.10, 112.23, 56.58, 42.41, 42.01, 39.82, 27.52, 26.99.

CARBON

methanol-d_3, 298 K

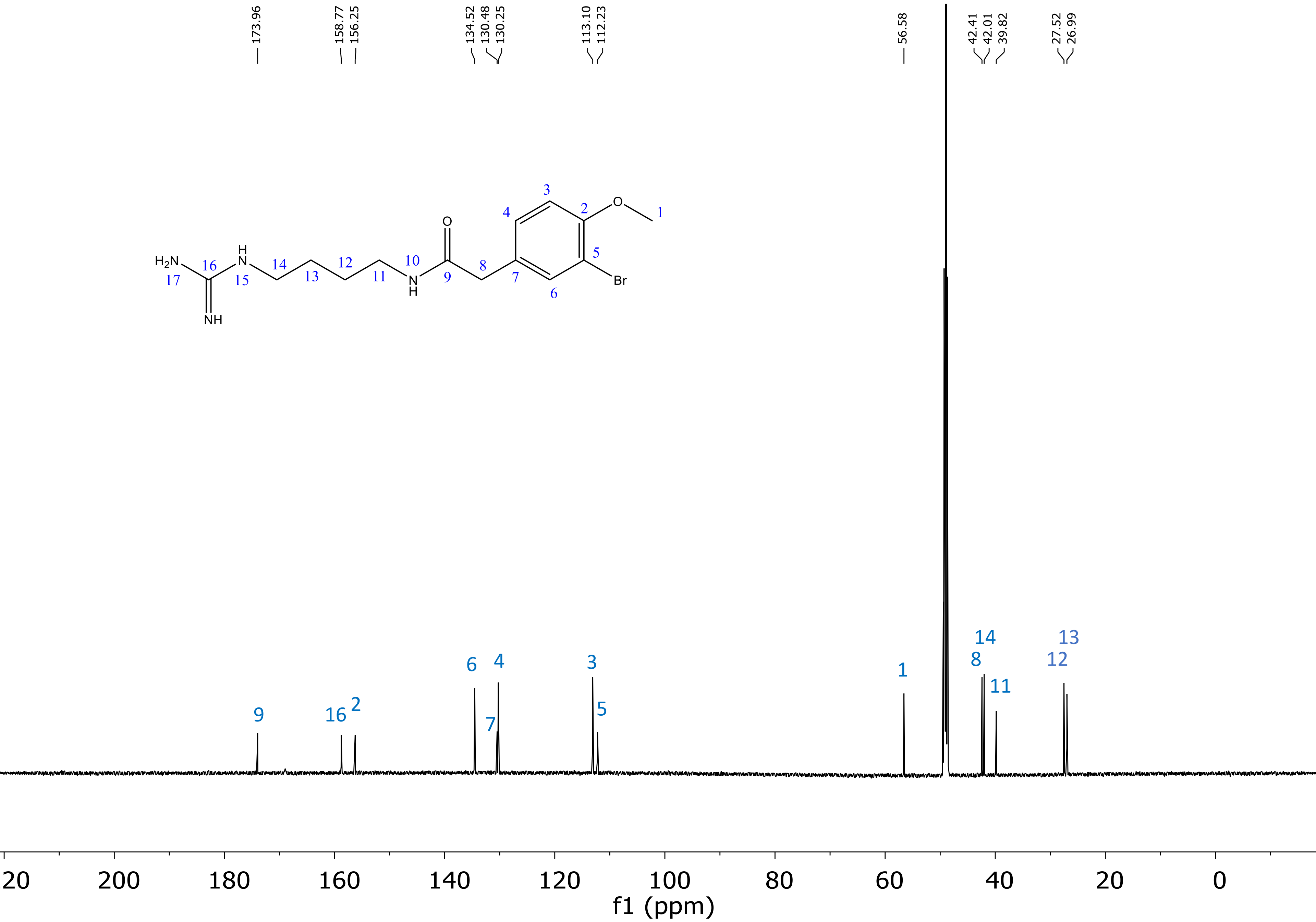

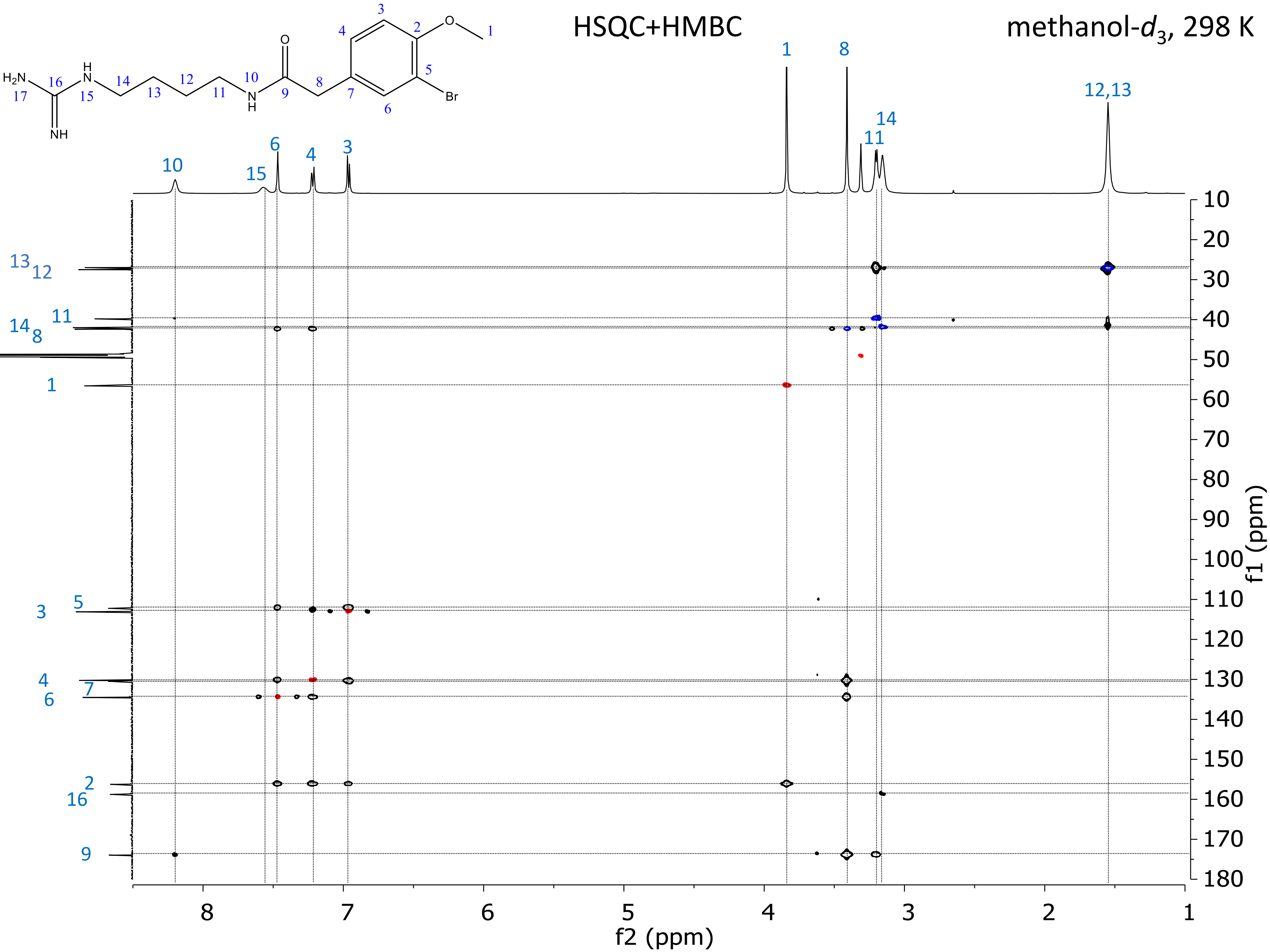
HSQC+HMBC
methanol-d_3, 298 K
f2 (ppm)
f1 (ppm)

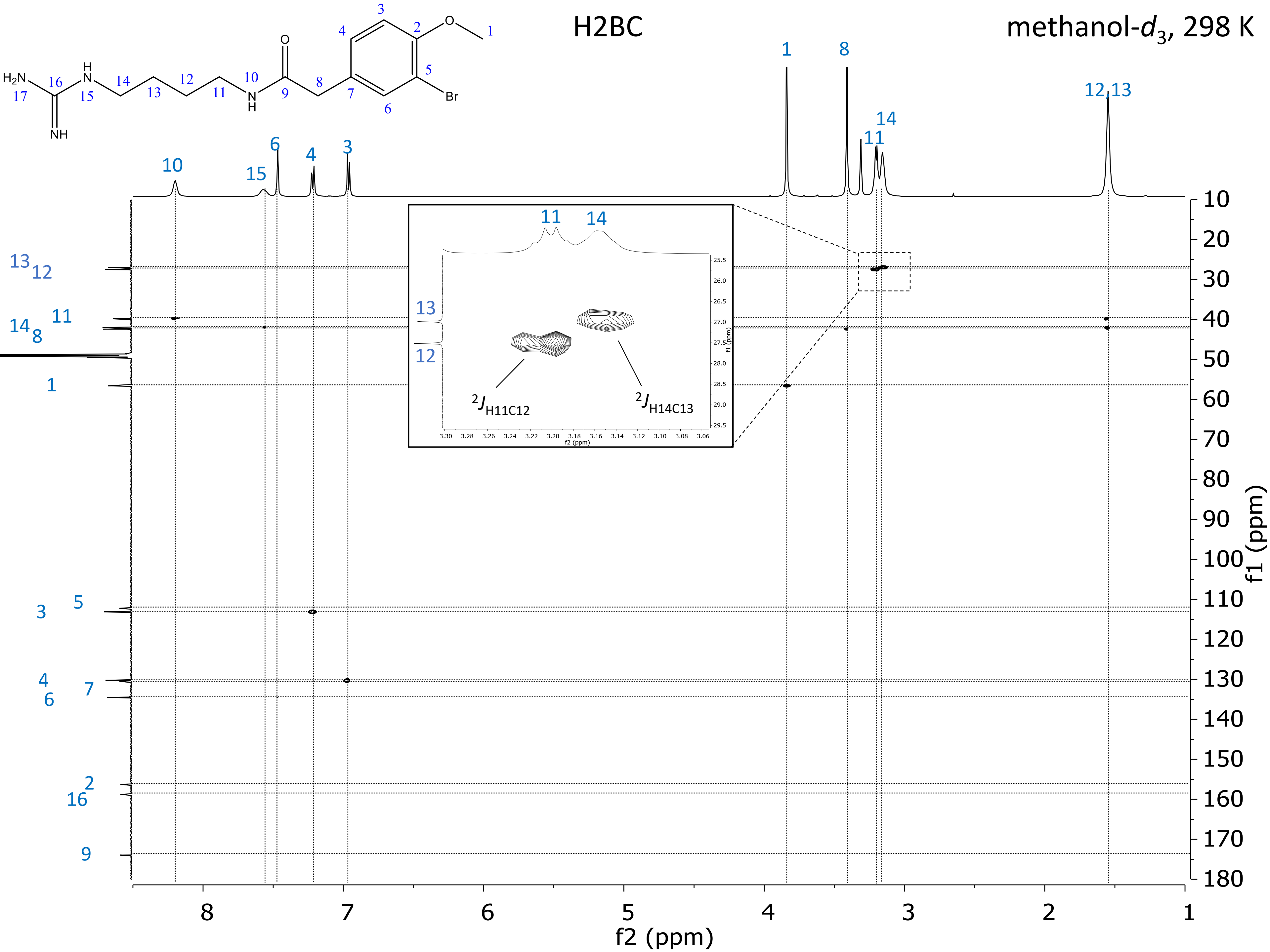
H2BC
methanol-d_3, 298 K
H_2N
NH
Br
O
1
8
14
11
12,13
10
15
6
4
3
13
12
14
8
11
1
5
3
4
7
6
2
16
9
$^2J_{H11C12}$
$^2J_{H14C13}$
f2 (ppm)
f1 (ppm)

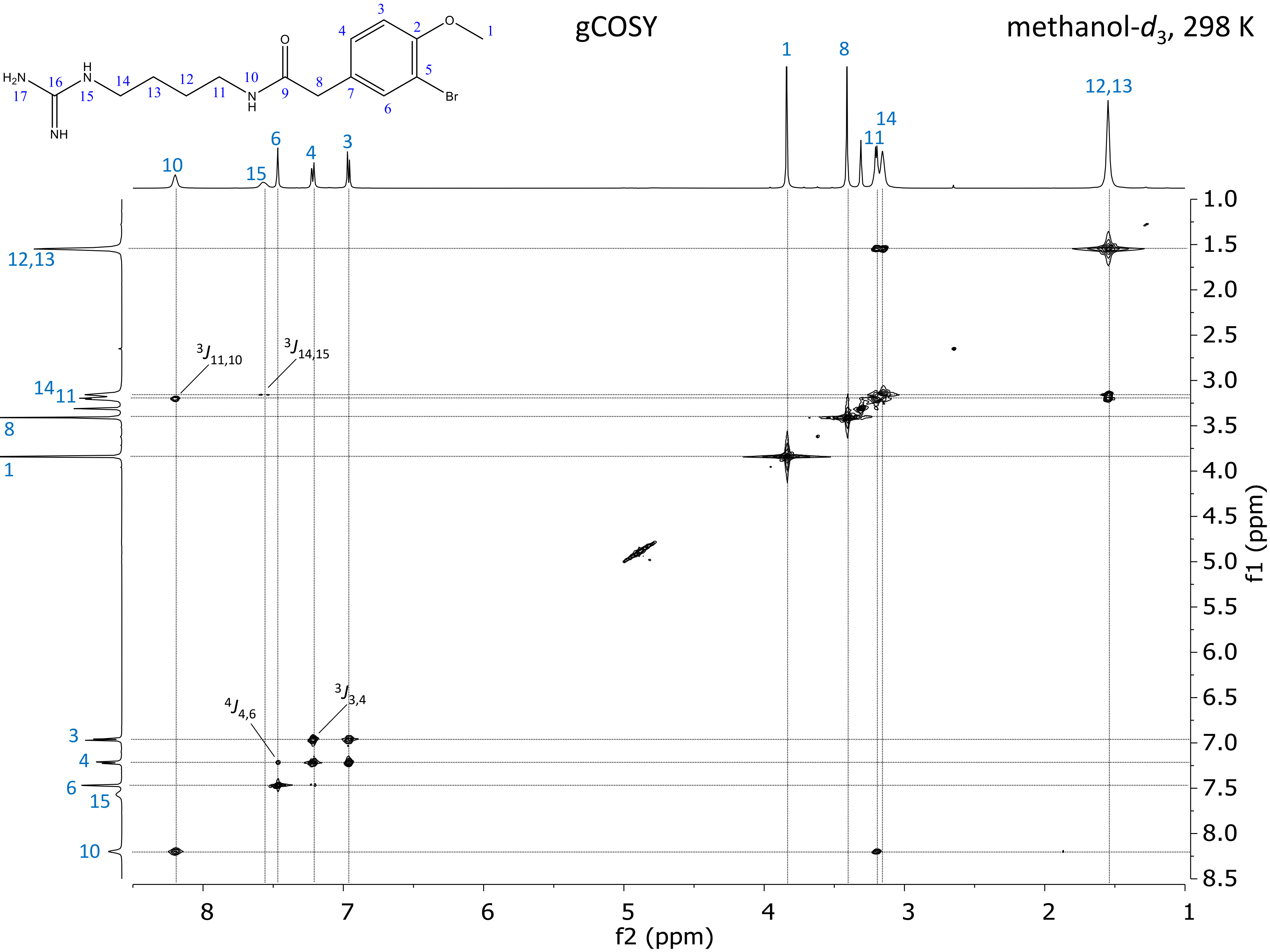
gCOSY
methanol-d_3, 298 K
$^3J_{11,10}$
$^3J_{14,15}$
$^4J_{4,6}$
$^3J_{3,4}$
f1 (ppm)
f2 (ppm)
Br
H_2N
NH

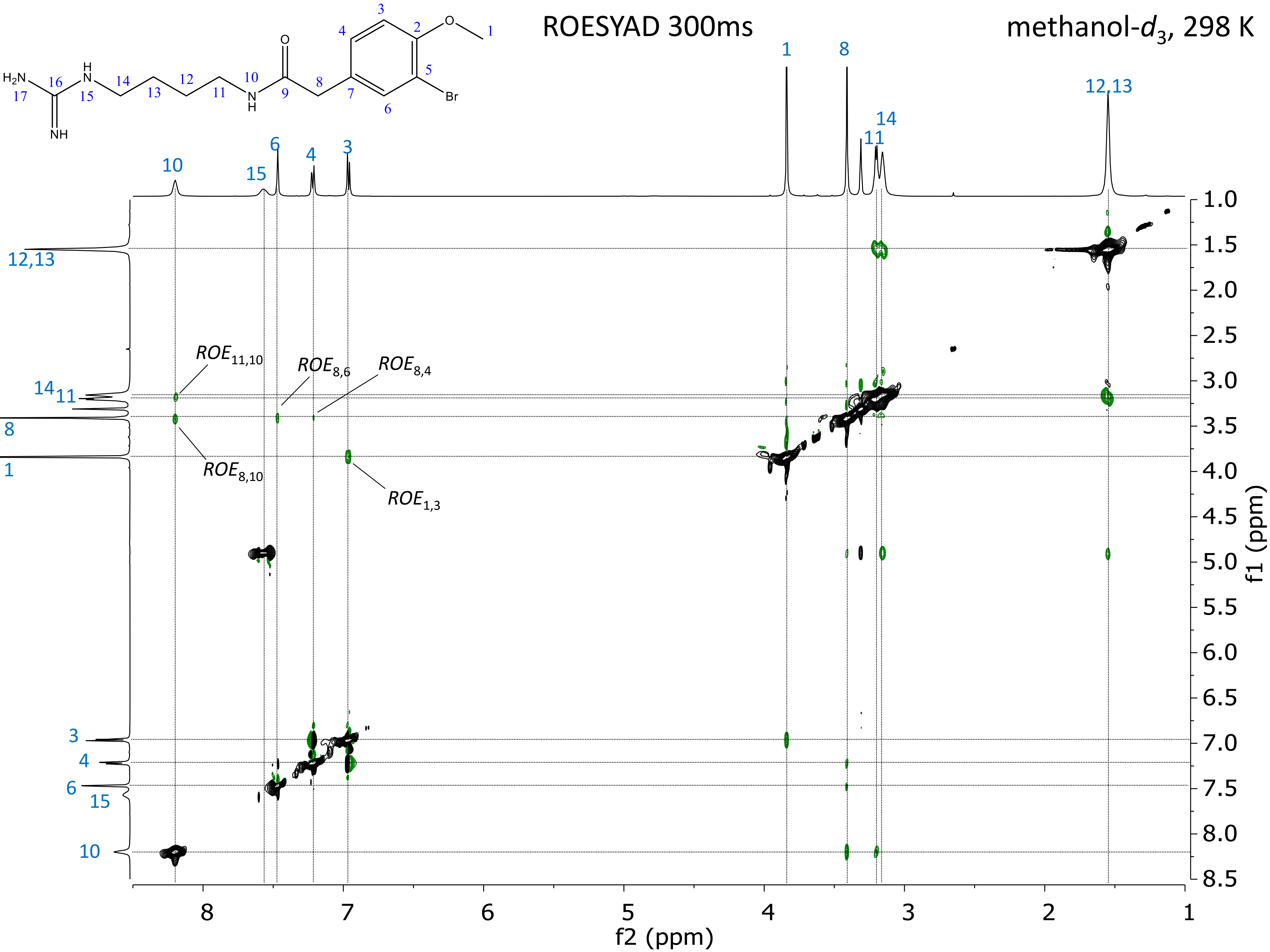
ROESYAD 300ms
methanol-d_3, 298 K
$ROE_{11,10}$
$ROE_{8,6}$
$ROE_{8,4}$
$ROE_{8,10}$
$ROE_{1,3}$
f2 (ppm)
f1 (ppm)
